# Keratinocyte Differentiation-Dependent Human Papillomavirus Gene Regulation

**DOI:** 10.3390/v9090245

**Published:** 2017-08-30

**Authors:** Sheila V. Graham

**Affiliations:** MRC-University of Glasgow Centre for Virus Research, Institute of Infection, Immunity and Inflammation, College of Medical, Veterinary and Life Sciences, University of Glasgow, Garscube Estate, Glasgow G61 1QH, UK; sheila.graham@gla.ac.uk; Tel.: +44-141-330-6256

**Keywords:** human papillomavirus, infection, epithelial differentiation, gene regulation, RNA processing

## Abstract

Human papillomaviruses (HPVs) cause diseases ranging from benign warts to invasive cancers. HPVs infect epithelial cells and their replication cycle is tightly linked with the differentiation process of the infected keratinocyte. The normal replication cycle involves an early and a late phase. The early phase encompasses viral entry and initial genome replication, stimulation of cell division and inhibition of apoptosis in the infected cell. Late events in the HPV life cycle include viral genome amplification, virion formation, and release into the environment from the surface of the epithelium. The main proteins required at the late stage of infection for viral genome amplification include E1, E2, E4 and E5. The late proteins L1 and L2 are structural proteins that form the viral capsid. Regulation of these late events involves both cellular and viral proteins. The late viral mRNAs are expressed from a specific late promoter but final late mRNA levels in the infected cell are controlled by splicing, polyadenylation, nuclear export and RNA stability. Viral late protein expression is also controlled at the level of translation. This review will discuss current knowledge of how HPV late gene expression is regulated.

## 1. Introduction

Over 210 human papillomavirus (HPV) genotypes have been characterized. Most HPVs do not cause any symptoms or disease. However, some can cause benign lesions such as warts on the hands or verrucas on the soles of the feet. Importantly, around 40 can infect the anogenital tract and cause benign lesions such as genital warts, or precancerous lesions such as cervical intraepithelial neoplasia [1]. Anogenital HPV infection is very common and affects around 80% of the population during their lifetime. Infection is usually transient because the virus is eventually recognized by the immune system and the infection is cleared without causing significant disease [2]. However, if infection with one of the so-called “high risk” (HR) anogenital-infective HPV genotypes becomes persistent, it can cause cellular changes that can lead to cancer formation [3]. HR-HPVs all belong to the alpha-papillomaviridae. There are 12 HR-HPVs for which the International Agency for Research on Cancer considers that there is sufficient evidence of carcinogenicity to humans (HPV16, HPV18, HPV 31, HPV 33, HPV35, HPV 39, HPV45, HPV51, HPV52, HPV56, HPV58 and HPV59). HPV68 is considered probably carcinogenic and HPV26, HPV53, HPV66, HPV67, HPV68, HPV70 and HPV73 are considered possibly carcinogenic [4]. The causative association of HPV infection with cervical cancer is well studied but more recently, HR-HPV has been found to be increasingly associated with oropharyngeal cancer, especially in younger men [5]. Understanding the infectious life cycle of HPVs that cause benign diseases—and of those HR-HPVs that can cause cancer—is essential for elucidating how persistent infection occurs and what molecular changes support cancer progression.

**Figure 1:**
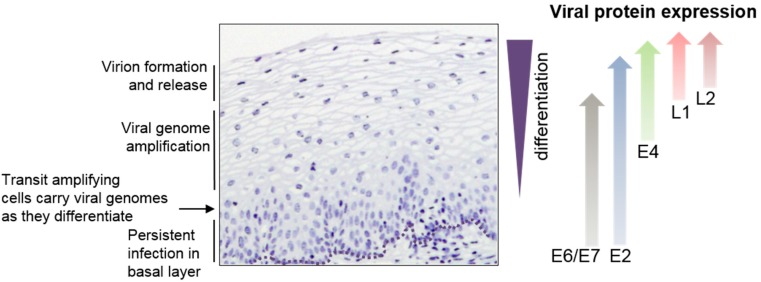
Eosin-stained HPV16-infected cervical epithelium (nuclei are stained purple). Key events in the viral replication cycle are noted on the left hand side. The approximate region of expression of viral proteins is shown on the right hand side. It is expected that E1 follows the pattern of E2 while E5 follows the pattern of E4. However, lack of suitable antibodies against these proteins have precluded confirmation of their expression pattern.

HPVs infect mucosal or cutaneous epithelia and there is a tight regulatory linkage between the viral life cycle and epithelial differentiation. Initial infection is targeted to dividing cells in the basal epithelial layer. Upon cell entry, the HPV genome is deposited in the nucleus and begins to express viral early proteins, which carry out an initial round of viral genome replication to yield around 50–100 genome copies per cell [6]. Once an infected basal epithelial cell divides, these viral genome copies are replicated and segregated equally into daughter cells. Continued presence of the viral genome over a period of several years in actively dividing epithelial cells results in persistent infection [6]. However, in a normal infection upon basal cell division, an infected daughter cell will become a transit amplifying cell that is destined to complete epithelial differentiation and move up through the various epithelial layers (Figure 1). During this process, there is a carefully orchestrated pattern of viral gene expression in response to epithelial differentiation such that different viral gene expression events are specifically linked to different epithelial layers (Figure 1). The viral genome within the cell nucleus responds to keratinocyte differentiation by activating viral late gene expression. Viral late proteins are required to accomplish productive viral genome amplification and virion production. These late events in the viral life cycle occur specifically in the differentiating upper layers of the epithelium (Figure 1) [7]. Therefore, cellular events linked to cellular gene expression make a significant impact on viral replication. It seems clear that persistent HPV infection is associated with a loss of the virus’s ability to carry out productive viral replication and synthesize new viral particles [8]. This review article will explore current knowledge of the viral and cellular molecular mechanisms of gene regulation that are required for the virus to accomplish late events in its life cycle.

## 2. Viral Genome Organization

**Figure 2:**
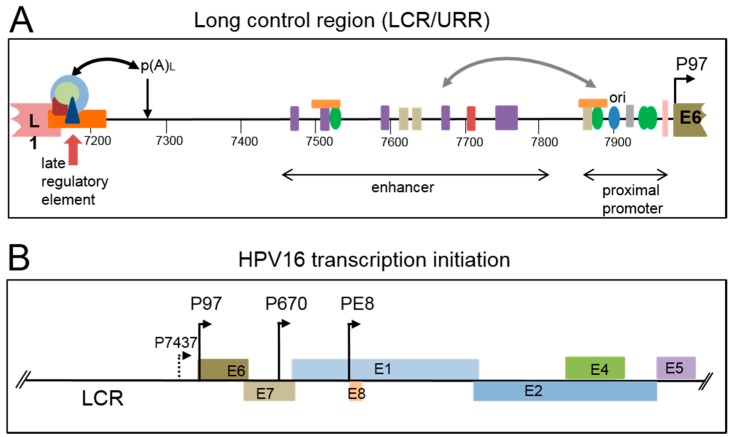
Regulatory elements on the HPV16 genome. (**A**) Details of the *cis*-acting elements, and the proteins that bind, located in the HPV16 long control region (LCR), otherwise called the upstream regulatory region (URR). The 3′ end of the L1 open reading frame is shown in pink. The 5′ end of the E6 open reading frame is shown in olive. The approximate positions of the proximal promoter and transcriptional enhancer are shown with double headed arrows. The origin of replication (ori) is marked with a blue oval. The late polyadenylation site is indicated with a downward facing arrow and p(A)_L_. The late regulatory element at the end of the L1 open reading frame is indicated with a red upward arrow and an orange box. The light blue circle indicates U1 snRNP, light green circle; SRSF1, dark red shape; U2AF, dark blue triangle; CUG-BP1 (see Table 1). The curved double headed black arrow indicates interactions between these factors and the polyadenylation complex (not shown). The viral transcription/replication factor E2 is shown as green ovals. Transcription factors are shown as rectangles. NF1; purple, Oct1; red, AP1; beige, Sp1; gray, YY1; orange. This is not an exhaustive list of transcription factors that bind the LCR. The curved double headed gray arrow indicates interactions between the enhancer and proximal promoter; (**B**) illustration of the LCR and early region of the HPV16 genome. Early open reading frames are shown as coloured boxes. The location on the genome of characterised promoters (P) is shown with arrows and associated numbers.

Non-enveloped icosahedral HPV virions are around 55 nm in diameter and each contains a double stranded circular DNA genome of around 8 kb in length. The genome can be divided into three regions, the early region containing open reading frames for at least seven viral regulatory proteins, the late region containing open reading frames for the two viral structural proteins, the capsid proteins, and an approximately 1 kb regulatory region called the Long Control Region (LCR) or Upstream Regulatory Region (URR) (Figure 2A). This non-coding region contains the viral early promoter and transcriptional enhancer, the viral origin of replication, the late polyadenylation site and the late (or negative) regulatory element (LRE/NRE) that controls late gene expression at various post-transcriptional levels (Figure 2A). The early viral promoter (HPV16: P97) is located at the 5′ end of the E6 open reading frame. Other start sites just upstream of this have been identified for HPV16 late mRNA in clinical samples (Figure 2B P7437) [9] and for HPV18 in raft cultured infected epithelial cells [10]. The viral late promoter (HPV16: P670) that is specifically activated in differentiated epithelial cells is located within the E7 open reading frame (Figure 2B). A third promoter, the E8 promoter, that is active both early and late in infection is located in the E1 open reading frame. Other possible promoters have been described but have not yet been fully tested for functionality (Figure 2B) [9,11,12,13,14].

### 2.1. Early Gene Expression

**Figure 3:**
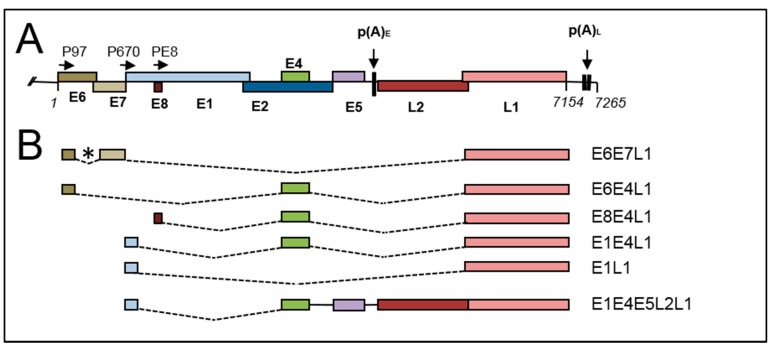
Splicing and polyadenylation of HPV16 late RNAs. (**A**) Diagram of the HPV16 genome. Coloured boxes indicate viral open reading frames. Promoters are indicated with arrows and (P). The positions of the early (pA_E_) and late (pA_L_) polyadenylation sites and indicated with downward arrows; (**B**) diagram of the main splicing events for generation of HPV16 late mRNAs. Boxes indicate open reading frames. Dotted lines indicate introns that are spliced out. (*), alternative splice acceptor sites can be used in the E6E7 gene region, including, for HPV16, sites at 409, 526 and 743. The putative coding potential of each mRNA is indicated to the right-hand side. This is a limited list of possible late mRNAs. A fuller list can be viewed at https://pave.niaid.nih.gov/#explore/transcript_maps.

The early promoter is responsible for expression of the early gene region. The viral replication factor E1 and its auxiliary protein E2, which is also the viral transcription factor, are probably the first proteins expressed during infection [15]. The viral E6 and E7 oncoproteins are also expressed early in a normal infection, but at very low levels to limit their oncogenic activity. However, in differentiating epithelial cells where cell division would normally be repressed, they act to stimulate cell cycle progression [16,17]. Moreover, because inappropriately dividing cells in the upper epithelial layers would normally be subject to apoptosis, E6 degrades p53 by targeting it for proteasome-mediated degradation, thus allowing HPV-infected cells to survive and support viral replication [18]. Other open reading frames in the early region encode E8^E2, an antagonist of the viral E2 replication and transcription factor [19], E4, which can remodel differentiated keratinocytes to allow release of progeny viral particles [20] and regulate the cell cycle [21], and E5, which has roles in keratinocyte signaling and immune evasion [22]. The proteins encoded by these open reading frames are expressed at highest levels in differentiating virus-infected cells [23] and they are required for productive viral genome amplification [24,25,26,27,28]. mRNAs encoding these proteins have been found to be expressed from both viral early and late promoters and this suggests that they may have roles at both the early and late stages of the viral life cycle. Early region mRNAs are polyadenylated at the early polyadenylation site which is situated just downstream of the E5 open reading frame (Figure 3A) [29].

### 2.2. Late Gene Expression and the Late Promoter

Late gene expression is initiated by the viral late promoter, terminated at the late polyadenylation site (Figure 3A) and occurs in the suprabasal layers (Figure 1) [11]. Viral late proteins E1, E2, E4 and E5 are required for vegetative viral genome amplification where the ~100 initially replicated viral genome copies are amplified to hundreds or thousands of copies. This is thought to take place using a rolling circle mode of replication, or recombination-dependent replication that is specific to this late life cycle stage [30,31] and is dependent upon the viral replication proteins E1 and E2. Expression of E2 appears to peak in the spinous layer of infected epithelia, co-incident with initiation of genome amplification [32,33,34]. Effective antibodies against E1 are lacking but it is likely that E1 expression follows a similar expression pattern to E2. HR-HPV E4, the most abundant viral protein, is expressed in the upper epithelial layers but cutaneous HPVs can express E4 in the basal layer [35]. E5 expression is likely also restricted to differentiated epithelial cells. Expression of major and minor capsid proteins L1 and L2 is always subsequent to E4 expression. However, each HPV genotype can display a distinct late gene expression program [35]. For example, mucosal HR-HPVs 16 and 31 express their capsid proteins only in the uppermost granular layer, but cutaneous HPVs such as HPV1 and HPV63 carry out late events much closer to the basal layer [35]. Importantly, HR-HPVs appear to restrict capsid protein synthesis to the uppermost layers [36,37,38]. This allows the virus to avoid activating the humoral immune response [2] and may facilitate the release of newly formed virions from disintegrating dead squames at the top of the epithelium.

Like many viral genomes, the genome organization of HPVs is complex. There are overlapping open reading frames (e.g., E2 and E4 or E1 and E8) and multicistronic transcription. Moreover, alternative promoter and polyadenylation site usage and alternative splicing yields many more mRNAs than there are open reading frames [39] and these seem to be essential for encoding the full complement of viral proteins. Therefore, although transcriptional regulation is important for the HPV life cycle and is a key factor in initiation of viral late gene expression in differentiating keratinocytes, much of the control of viral gene expression occurs at a post-transcriptional level.

## 3. Viral Late Promoter Activity

Viral late promoter activation is a key mechanism behind the initiation of late gene expression. The mucosal oncogenic virus late promoters (HPV16 P670 [36], HPV18 P811 [40] and HPV31 P742 [41]) are the best characterized but similarly positioned promoters exist for other HPVs including the cutaneous HPV5 (P840) [42], and HPV8 (P7535) [10,43,44]. The HPV31 late promoter has over 30 possible initiation sites stretching from genome positions 605 to 779 [41,45] while for HPV16, transcription initiation sites have been mapped to various nucleotides within 200 nucleotides surrounding P670 [11,36]. HPV31 possesses two differentiation-responsive elements in the late promoter region [41] and a number of transcription factors have been shown to bind and regulate the HPV16 and 31 promoter regions [46,47]. The HPV18 late promoter is likely also regulated by similar differentiation-dependent transcription factors but this has not been fully elucidated. A recent intriguing observation is that HPV18 late promoter activity is controlled by the orientation of viral DNA replication, thus providing a clear link between vegetative viral DNA amplification and late transcription [40]. Moreover, this study revealed that hnRNP proteins A/B and DOB bind a transcriptional repressor to regulate late gene expression. As well as promoter control by proximal elements, it has been shown that the HPV31 late promoter is also controlled by the viral enhancer located in the LCR [41]. Linked to this observation, recently it has been shown that the promoter is mainly controlled at the level of transcription elongation. Although RNA polymerase II is bound at the late promoter in undifferentiated keratinocytes, elongation is inefficient and does not progress to the late region. In contrast, in differentiated cells, the enhancer recruits members of the BET family proteins—including Brd4 and its binding partner CDK8—to the Mediator complex to stimulate transcription elongation [48]. Changes in signaling during keratinocyte differentiation must also regulate the late promoter, for example PKCδ [49] has been shown to activate it. Finally, the E8 promoter, responsible for expression of the replication and transcriptional repressor E8^E2, is active at both early and late phases of the life cycle but its regulation has not been extensively characterized as yet [50].

There is evidence of a reciprocal coordination between activity of the viral early and late promoters, because the early E7 protein has been shown to control the HPV16 late promoter [51]. Although it has been proposed that early oncoprotein activity wanes as the late phase of the life cycle gets underway [23,52], transcripts encoding E6 and E7 have been detected in the cytoplasm of cells in the mid-upper epithelial layers [53,54,55,56]. Indeed, it has been reported that transcription initiation at the HPV31 early promoter is not down-regulated by differentiation [13] and HPV31 late transcript mapping in differentiated keratinocytes detected transcription initiation in the vicinity of the early promoter giving rise to several RNAs encoding E6 and E7 [57]. It is unlikely that RNA polymerase could load simultaneously onto the three closely spaced promoters (Figure 2B) that are active at late stages in the viral life cycle. Although it is possible that a stochastic choice of promoters takes place, there must be differentiation-specific control exerted. Therefore, polymerase loading and/or rate of elongation, controlled by transcription complexes recruited in a differentiation-specific manner to the viral enhancer and promoter regions, is a more likely mechanism to ensure inter-dependent promoter activity at late times during the viral replication cycle.

## 4. Differentiation-Dependent Regulation of Polyadenylation

Polyadenylation is a two-step process involving cleavage at the cleavage site and subsequent addition of 200–250 A residues to the 3′ end of the mRNA [58]. Polyadenylation is essential for mRNA export from the nucleus to the cytoplasm, mRNA stability and translation. Mammalian polyadenylation sites comprise two cis-acting regulatory elements, an upstream A(A/U)UAAA element, located 10–30 nucleotides upstream of the cleavage site, that binds the cleavage and poyadenylation specificity factor (CPSF). A 73 kDa subunit of CPSF possesses endonuclease activity for the cleavage reaction, but CPSF is also required for poly(A) addition. A GU-rich element, followed by a UUU motif, located 10–30 nucleotides downstream of the cleavage site binds the cleavage stimulatory factor (CstF). CstF is required for cleavage and it stabilises the binding of CPSF upstream through protein-protein interactions between its 77 kDa subunit and the 160 kDa subunit of CPSF. 

The exact sequence of the cis-acting elements to which these protein complexes bind determines whether polyadenylation is efficient or not by controlling the stability of the polyadenylation complex [58]. HPV genomes possess at least two polyadenylation sites—the early site (p(A)_E_) situated in the early 3′ untranslated region (UTR) (Figure 3A) downstream of the E5 open reading frame, and the late site, p(A)_L_, located in the 5′ end of the LCR [59]. Early trancription terminates at the early site, while late mRNA synthesis uses the late site [36,37,60,61]. There is good evidence for differentiation-dependent control of both sites. Because late mRNA production initiates from the late promoter located in the early region, transcription must proceed through the early polyadenylation site to synthesize late mRNAs. This suggests that either rate of RNA polymerase passage through the early polyadenylation site is up-regulated or use of the early polyadenylation site is repressed at late times of infection. The HPV31 early polyadenylation site contains a consensus CPSF binding site, but each of three possible CstF binding sites downstream are of weak consensus and lead to heterogeneity in polyadenylation site usage [62,63]. The HPV16 early polyadenylation site has a similar organisation but also contains a long U-rich stretch upstream of the CPSF-binding motif that binds auxilliary polyadenylation factors such as hFip 1, to enhance early polyadenylation [64]. Other HPV early polyadenylation sites are also of weak consensus [65], however, the HPV18 early site appears to contain good consensus polyadenylation signals in addition to several upstream U/GU-rich motifs that could regulate efficient polyadenylation [10]. The HPV31 late polyadenylation site has a simple AAUAAA and downstream G/U rich element organisation [66]. HPV16 uses two tandem late polyadenylation sites [11], the first of which is of weak conensus and used less frequently, while the second is of strong consensus [11,67]. HPV18 has a single late AAUAAA site and a downstream GU-rich motif but transcript cleavage was detected at a range of nucleotides over a 35 nt region [10].

### 4.1. Repression of the Early Polyadenylation Site and Late Gene Expression

Most studies have arrived at the consensus view that late mRNA expression is controlled largely through negative control of the early polyadenylation site, thus allowing transcription to proceed into the late region. This may be the reason why the early polyadenylation site has to be inherently weak to allow fine control by RNA binding proteins. The role of the early polyadenylation site in the control of late gene expression was first demonstrated for HPV31. Mutational analysis of the early polyadenylation site revealed that the early CPSF-binding site was a key inhibitor of late transcript production [63]. However, this study also uncovered an element in the 5′ end of the downstream L2 open reading frame that repressed capsid mRNA production, probably via positive enhancement of the early polyadenylation site through binding the 64 kDa subunit of CstF (CstF64) [62,63]. Subsequently, a similarly located element was discovered for HPV16, but in this case the element bound not only CstF64 but also hnRNP H through multiple GGG motifs [65,68]. It has been proposed that these motifs cause formation of a specific RNA secondary structure that is optimal for interaction of RNA binding proteins with the upstream polyadenylation complex. Whether these would be stimulatory or inhibitory interactions in vivo is not known.

These important findings indicate that the “weak” early viral polyadenylation site could be enhanced by upstream and downstream sequences that could bind polyadenylation-stimulatory factors and thus facilitate polyadenylation of early mRNAs. Alternatively, cellular factors binding to RNA motifs in the vicinity of the polyadenylation site could create secondary structures that are suboptimal for efficient early polyadenylation leading to stimulation of late gene expression. Such cellular factors could be expressed in a differentiation-dependent manner. Polyadenylation regulation, via the carboxyl terminal domain of RNA polymerase II, is coupled to other post-transcriptional events, including RNA capping and splicing, all of which take place co-transcriptionally [69]. Therefore, HPV RNA splicing could be expected to regulate polyadenylation. As shown in Figure 3B, HPV mRNAs are the products of a range of splicing events. The splicing regulatory protein Serine Arginine Splicing Factor 3 (SRSF3) binds a splicing enhancer element in the E4 open reading frame to facilitate polyadenylation at the early polyadenylation site [70]. Another E4 element binds SRSF1 and appears to favour synthesis of HPV16 mRNA early polyadenylation [71,72]. Splicing factors are known to control polyadenylation by forming protein-protein interactions across exons that link to downstream polyadenylation complexes [58,73]. Differential expression of factors controlling the switch from early to late polyadenylation during keratinocyte differentiation would be necessary to induce repression of the early polyadenylation site. However, there is controversy regarding differentiation-stage-specific expression of splicing and polyadenylation factors because different patterns are observed in uninfected versus HPV-infected or HPV-associated tumour tissue [70,74,75]. Future studies should analyse protein expression in normal human cervical keratinocytes and the same cells transfected with episomal HPV genomes. Finally, HPV E2 has been implicated in a general, potentially low level, inhibition of polyadenylation by inhibiting assembly of the CPSF complex [76]. Rising levels of E2 in the mid to upper epithelial layers could lead to selective repression of early gene expression because the majority of mRNAs are polyadenylated at the early polyadenylation site. Early polyadenylation repression would lead to increased transcriptional read-through from the early region to the late region and a corresponding increase (despite some inhibition of late polyadenylation) in production of HPV16 late mRNAs [76]. It remains to be tested whether other E2-interacting proteins, both viral (e.g., E4, E1, E8) and cellular (e.g., SR proteins) [77,78], might modulate the effects of E2 on polyadenylation.

### 4.2. Control of Late Gene Expression Occurs at Multiple Post-Transcriptional Levels

**Figure 4:**
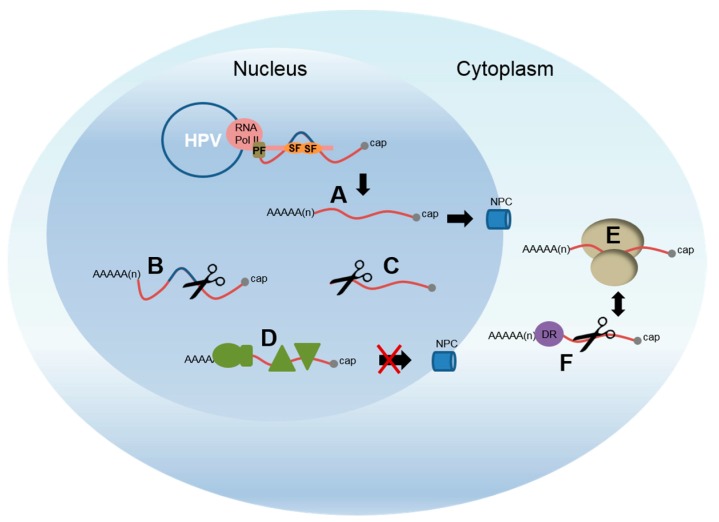
Cartoon of HPV gene regulation in the nucleus and cytoplasm. Transcription of the HPV genome (blue circle) is carried out by RNA polymerase II (pink oval). The carboxyl terminal domain of the polymerase (pink thin rectangle) acts to integrate transcription and RNA processing by binding splicing factors (SF; e.g., SRSFs and hnRNPs, orange ovals) and polyadenylation factors (PF, olive rectangle). These are then able to bind and process the nascent RNA molecule as it emerges from the RNA polymerase exit channel. A. Fully processed mRNAs are able to be exported from the nucleus to the cytoplasm through nuclear pore complexes (NPC, blue tubes) to access the ribosomes (beige ovoids) for translation (E.). Aberrantly formed mRNAs are targeted for decay. B. mRNAs that aberrantly retain introns. C. mRNAs that are not properly polyadenylated, or capped. D. RNAs that bind protein complexes (olive shapes) that inhibit nuclear export. F. RNA decay factors (DF, purple circle), especially those that bind 3′ untranslated region motifs, can control levels of mRNAs in the cytoplasm linked to translation. This is not an exhaustive list of possible control mechanisms.

Several different mechanisms of regulating capsid protein expression have been reported. These include transcription initiation and elongation—and polyadenylation, as discussed above—but also splicing, mRNA stability and translation. These processes are all linked in the cell (Figure 4). Transcription and RNA processing is linked through the carboxyl terminal domain of RNA polymerase II [69], while mRNA stability and translation on the ribosomes are also intimately connected [79]. Cell signaling regulates each of these pathways in order that cells can direct expression of the appropriate set of mRNAs and proteins to ensure normal function. An early study revealed that protein kinase C (PKC) signalling post-transcriptionally controlled late protein expression in HPV31-infected keratinocytes [61]. The mechanism of action could be through polyadenylation, splicing or RNA stability control. The following sections will discuss each of these post-transcriptional mechanisms in turn. However, it is important to remember that changes in one process will impact on the others and alterations in differentiation-specific expression levels of cellular proteins will also play a major role.

### 4.3. Control of Late Polyadenylation

The HPV late polyadenylation sites that have been analysed thus far are predicted to be used efficiently because they possess excellent consensus *cis*-acting control sequences. However, polyadenylation efficiency can be positively or negatively controlled through upstream sequence elements (USEs) or downstream sequence elements (DSEs). These elements are usually U-rich and are found in the vicinity of some cellular—e.g., COX-2 [80], human complement factor C2 [81] and collagen genes [82]), and many viral, polyadenylation sites (e.g., HIV [83], SV40 [84,85] and adenovirus [86]. They work by recruiting RNA-binding proteins that can stimulate or stabilise the polyadenylation complexes perhaps by inducing secondary structures that facilitates appropriate protein-proteins interactions. The upstream and downstream *cis*-acting elements that regulate HPV16 and HPV31 early polyadenylation site usage appear to work in this way. Not all USE/DSEs stimulate polyadenylation. For example, U1A RNAs possess a 3′UTR USE that binds U1A protein to negatively autoregulate its expression [87].

Upstream negative regulatory elements that control late gene expression have been identified in a wide range of HPV genomes [88]. These cis-acting U-rich elements are between 50 and 100 nts in length and are positioned at the 3′ end of the L1 coding region and span into the late 3′ untranslated region (3′UTR) (Figure 2A) [89]. They can bind a number of RNA-binding proteins that have known functions in splicing, polyadenylation and mRNA stability (Table 1). In particular, the HPV16, HPV18 and 31 elements contain 5′ splice sites that could bind U1 snRNP [10,66,90]. U1snRNP is a large RNA-protein complex which, when bound upstream of the polyadenylation site, could repress formation of the polyadenylation complex. Indeed, there is direct evidence that the HPV16 element inhibts HPV late polyadenylation in HeLa cells that possess a basal epithelial cell phenotype [91], and this could be another mechanism of ensuring restriction of capsid protein expression to differentiated kertatinocytes. Other HPV late elements (including HPV16) are more similar to AU-rich elements (AREs) that control mRNA stability [88] and theiractivity may be mediated by differentially-expressed cellular proteins such as the mRNA stability regulator, HuR [89,92].

### 4.4. Regulation of Late RNA Stability

RNA stability is a very significant layer of control in gene expression because degradation or stabilisation of a transcript can rapidly alter its cellular levels (Figure 4) [93]. In the nucleus, incorrectly processed or mutant RNAs, detected by virtue of the RNA-binding proteins that are loaded upon them, are destroyed by the exosome and therefore are not exported to the cytoplasm for translation [94]. In the cytoplasm, mRNAs can be stabilised or destroyed by an AU-rich element (ARE)-mediated pathway or by nonsense-mediated decay (NMD) [93,95].

Evidence from early in situ hybridisation studies of mucosally-infective low risk HPV6, 11, and high risk 16, 18 and 31-positive lesions revealed that some late region transcripts were detected in mid-epithelial layers but that cytoplasmic late mRNAs, i.e., fully formed mRNAs that are able to be exported from the nucleus to the cytoplasm, were detected only in the upper epithelial layers [53,54,55,56]. This suggests that although the late region can be transcribed in less differentiated cells, RNA processing or export from the nucleus is inhibited, or cytoplasmic late mRNAs are highly unstable. Late pre-mRNAs may be synthesised in less differentiated keratinocytes, at least in the W12 and NIKS16 models of the virus life cycle, because it is possible to induce L1 protein expression by manipulating levels of certain RNA-binding proteins [92,98]. Moreover, expression of reporter constructs containing the entire wild type HPV16 late regulatory element gave significantly increased RNA nuclear retention compared to similar constructs with mutations affecting key protein-binding regions of the element [97]. This suggests that RNA-protein interactions on the late regulatory element in undifferentiated cells could inhibit late pre-mRNA processing or nuclear export, correlating with the previous in vivo observations [53,54,55,56]. Therefore, nuclear retention of late transcripts synthesised in undifferentiated keratinocytes could be a direct result of polyadenylation inhibition via the late regulatory element.

The cutaneous HPV1 and mucosal HPV16 late regulatory elements have been shown to bind the RNA stability regulator HuR [92,97,99,100,101]. The HPV1 element also binds the RNA stability regulator hnRNP C1/C2 [97,102]. Regulation of mRNA turnover could control capsid protein expression. For example, HuR overexpression in undifferentiated keratinocytes caused the induction of L1 protein expression, while siRNA-mediated inhibition in differentiated keratinocytes caused loss of L1 expresion [92]. Similarly, the HPV16 L2 open reading frame was found to contain a 5′and a 3′ regulatory element that acts at the level of mRNA stability. The 5′ element negatively regulated CAT reporter mRNA stability by 3-fold in the cytoplasm of HeLa cells expressing the HIV *tat* protein, while the 3′ element was only weakly active [103]. Interestingly, the HPV1 L2 gene does not appear to contain such an element, or indeed, any major regulatory element in the late gene region, apart from the 3′UTR element [103].

### 4.5. Late RNA Splicing

Late mRNAs are polycistronic transcripts containing the 1.5 kb L1 open reading frame with several different possible upstream spliced exons (Figure 3B). Other late mRNAs contain both L1 and L2 (1.4 kb) open reading frames in a read-through mRNA that also contains the short E4 (274 bps) and E5 (236 bps) open reading frames (Figure 3B). Given that the average size of a eukaryotic terminal exon is 627 nts [104], this means that the terminal exons of these mRNAs are unusually long at either 1.5 or 2.9 kb. A process called exon definition is important for efficient splicing, especially of long exons [73] and exons are defined by protein complexes formed at the 5′ and 3′ exon-intron boundaries and by splicing regulatory factors such as SR proteins and hnRNP proteins bound to exon- and intron-internal cis-acting sequences [105]. Chromatin conformation and RNA secondary structure also play a role in determining efficiency of splicing and which exons are spliced out or retained [105]. Moreover, due to coupling of transcription elongation and RNA processing—including splicing (Figure 4)—the promoter used to initiate gene transcription can influence splicing efficiency [106]. Although exon size is often greatest in genes with few introns (late mRNAs have only one or two introns) [107], the cellular RNA processing machinery would be predicted to process the late RNAs inefficiently. The late mRNAs are generated by splicing from four possible splice donor sites, one in the E6 region (HPV16 nt 226, HPV18, nt 233, HPV31 nt 210), one in the E8 gene region (HPV16 nt 1301, HPV18 nt 1202) and one at the end of each of the E1 and E4 open reading frame (HPV16 nt 3632, HPV18, nt 3696, HPV31, nt 3590) (Figure 3B). The latter two splice events are by far the most frequently used. At least for HPVs 16, 18 and 31, late RNAs can be initiated at both the early and late promoters, although the relative abundance of early promoter-initiated late RNAs versus late, or E8, promoter-initiated late RNAs has not been determined in the different layers of the infected epithelium.

### 4.6. Control of Late mRNA Splicing

In general, cellular and viral splicing is positively controlled by SR splicing factors [108], and negatively by hnRNP proteins [109,110]. HPV controls expression of SRSF1, 2 and 3 through the viral E2 transcription factor [98] and SRSF levels peak in the mid to upper layers of HPV-infected epithelial [75,98] where late gene expression commences [23], and E2 is most highly expressed [32,33]. Recently, we reported that SRSF3 controls L1 protein expression in a differentiation-dependent manner. siRNA depletion of SRSF3 in differentiated HPV16-positive keratinocytes inhibited expression of the capsid mRNA and protein while SRSF3 overexpression in undifferentiated epithelial cells caused the induction of L1 protein. Levels of the E1^E4^L1 mRNA were undetectable in differentiated keratinocytes treated with siRNA agaisnt SRSF3. While there was a corresponding increase in levels of the read-through L2L1 mRNA, it is likely that loss of SRSF3 affects other RNA processing events as well as splicing. We also found that SRSF1 contributed to control of capsid protein expression [98]. This is in agreement with a previous study, which demonstrated that SRSF1 contributed to regulation of the major alternatively spliced E4^L1 mRNA and the L2L1 read-through late mRNA (Figure 3B) [72]. A third SR protein, SRSF9 has also been shown to enhance splicing of late RNAs [111].In HeLa cells, SRSF9 inhibited splicing at the splice acceptor site at the 5′ end of the E4 open reading frame. This led to use of the next downstream splice acceptor site located at the 5′ end of the L1 open reading frame resulting in enhanced L1 mRNA production.

The L1 open reading frame contains a splicing enhancer element that can be repressed by hnRNP A1 in HeLa cells [112,113]. However, hnRNP A1 appears to be upregulated during differentiation of HPV-infected keratinocytes [114]. Although this could be counter-balanced by increased expression of splicing enhancer proteins—such as SRSF1—that negate the effects of hnRNP A1, it will be important in future to try to understand the complexity of late RNA splicing regulation in differentiated keratinocytes. hnRNP H can bind a G-rich element in the L2 coding region and, as discussed in Section 4.1, can control early polyadenylation [65]. The importance of the early polyadenylation site usage in preventing late mRNA expression means, in effect, that hnRNP H can also inhibit late mRNA expression. Other sequences in the viral early region can control late mRNA expression. hnRNPs A2/B1 and D inhibit L1 mRNA production by binding an element in the E4 open reading frame that likely suppresses use of its 3′ splice donor site to inhibit splicing to the L1 splice acceptor site [115]. Similarly, hnRNP I, (polypyrimidine tract binding protein (PTB)) can induce late transcript expression by inhibiting the same splice donor site [116]. Conversely, overexpression of hnRNP C1, and another hnRNP protein, RALYL, induced late mRNA production in C33A cervical cancer cells. hnRNP C1 binding to the early 3′UTR was shown to activate L1 mRNA expression by activating the E4 splice donor site [117]. No doubt the work published thus far only scratches the surface of the possible SR and hnRNP regulatory interactions that can control HPV late gene expression. Most studies have examined HPV16. Therefore, it will be of interest to compare the data from this HR-HPV with that from other HPV types, for example another HR-HPV such as HPV31 or 18, the low risk anogenital infective HPVs 6 and 11, or the cutaneous infective HPV1. In fact, some elements that control late gene expression have been shown to be present in a range of HPV types [88,118] suggesting that such an important regulatory event is likely to be conserved across different HPV types. Finally, it is important that differentiation is taken into account in such studies due to possible HPV infection-associated changes in the various proteins that can control splicing.

### 4.7. Control of Capsid Protein Translation

Control of the translation of viral late proteins is still poorly understood. HPV polycistronic late mRNAs are predicted to be inefficiently translated to yield capsid proteins. This is because of the Kozak rules of translation initation that predict that subsequent open reading frames in polycistronic transcripts will be poorly translated [119]. In the scanning model of translation initiation, the ribosome recognises the first AUG in an mRNA as the start codon. Moreover, translation is inhibited when an intron, that often contains pseudo splice sites, is retained in the mRNA, as it will be for L2L1 readthrough RNAs. The first translation start codon in most late mRNAs is at the start of the E1 open reading frame because the E4 open reading frame does not possess an AUG codon; this is donated by splicing. E4 is the most abundant viral protein [23], suggesting that it is very efficiently translated. The L2 and L1 open reading frames are downstream of E4 in all mRNAs indicating inefficient translation. L1 protein is indeed much less abundant than E4 in HPV-infected tissues [120], and although levels of L2 relative to E4 have not been directly observed, it is likely that L2 is expressed at an even lower level than L1 because only one molecule of L2 is required for every 5 molecules of L1 in the virus capsid [120]. If the capsid protein mRNAs were particularly abundant, this might facilitate capsid protein expression, and they do seem to be readily detected in HPV-positive lesions [53,54,55,56]. Alternatively, it has been proposed that viral translation efficiency could be assured through use of rare codons that are less frequently used for cellular mRNA translation [121,122]. HPV genomes possess a high A+T frequency at the 3rd position in codons in the L2 and L1, but not the E4, open reading frames [122]. At least in experiments using reporter gene constructs, this seems to determine the keratinocyte differentiation-specific expression of L1 protein [123,124]. Undifferentiated and differentiated keratinocytes were found to have a different tRNA profile whereby only differentiated keratinocyte tRNAs allowed efficient L1 translation [123,125]. This regulatory mechanism was favoured in G2/M-like growth arrested cells that expressed keratinocyte differentiation markers [126]. Therefore, the tRNA pools available in differentiated keratinocytes may greatly favour translation of HPV late mRNAs.

Other studies have examined whether late mRNA-binding proteins control translation. The HPV16 L2 open reading frames contains a 3′ element that binds hnRNP K and poly (rC) binding protein (CBP) [127]. Depletion of these proteins in an in vitro system caused a reduction in L2 capsid protein production but a definitive role for the RNA binding proteins in translation, as opposed to other events—for example mRNA stability—was not tested. Indeed, the L2 open reading frame has a second inhibitory element located within the first 845 nucleotides that acts to destabilise the mRNA, and therefore to inhibit its translation [103]. Any of the late regulatory elements discussed above that may regulate late mRNA processing are also possible regulators of late mRNA translation because they bind cytoplasmic proteins that can affect mRNA stability [89]. For example, the HPV1 late regulatory element has five U-rich motifs that render the late mRNAs unstable in HeLa cells and this results in inefficient translation [128].

The above mechanisms may all play a part in facilitating viral capsid protein expression from late mRNAs. However, as stated previously, these mRNAs are unusual in structure due to their polycistronic nature and this could exacerbate recognition of appropriate start codons for translation initiaiton. For example, in the case of the L1 open reading frame, there is always at least one AUG start codon prior to the L1 start. For L2 in the L2L1 read-through RNA there are at least three start sites that the ribosome must pass through in order to translate L2. Perhaps RNA-binding proteins obscure the upstream intiation codons in some way during translation initiation. Alternatively, other putative promoters have been described for a number of HPVs that could give rise to monocistronic mRNAs encoding each of the late proteins separately [7,11]. However, the activities of these have not been examined in the context of the viral life cycle.

## 5. Conclusions

This review has aimed to highlight the various regulatory mechanisms that control late gene expression of human papillomaviruses. We have most information on the regulation of late gene expression for HR-HPVs and it cannot always be assumed that other HPVs will undergo similar regulatory controls. Over the last decade, the extent of the association of the HPV replication cycle with keratinocyte differentiation has become clearer. Future studies on HPV gene regulation should continue to focus on how differentiated keratinocytes control viral capsid production. In particular, what differentiation-specific changes in transcription factors and RNA-binding proteins might be relevant to facilitating viral late mRNA production and late protein translation should be worked out. Understanding the mechanism of control of capsid protein expression could facilitate the design of antivirals to induce expression of the highly immunogenic capsid proteins inappropriately in the lower epithelial layer to enhanced immune recognition and viral clearance.

## Figures and Tables

**Table 1:** RNA-binding proteins that interact with RNA regulatory elements in the late 3′UTR of different HPVs [96].

HPV Genotype	Protein	Function
HPV1	HuR	mRNA stability, polyadenylation, nuclear export
	hnRNPC1/C2	Splicing, polyadenylation, mRNA stability, nuclear export
	PABP	Polyadenylation, translation
HPV16	U1snRNP	splicing
	HuR ^1^	mRNA stability, polyadenylation, nuclear export
	SRSF1 ^3^	Splicing regulation
	hnRNP A1	Splicing regulation
	U2AF	Auxiliary splicing factor
	CstF-64	Polyadenylation
	CUG-BP	Alternative splicing, translation
HPV31	U1snRNP ^2^	splicing
	HuR	mRNA stability, polyadenylation, nuclear export
	U2AF	Auxiliary splicing factor
	CstF-64	Polyadenylation
HPV18	U1snRNP^2^	splicing

^1^ HuR binding was found by our group [92,97], but not by others [88,91]; ^2^ U1snRNP binding is inferred due to the presence of 5′ splice sites, but not proven; ^3^ Binding is indirect.
